# Opioids, Polypharmacy, and Drug Interactions: A Technological Paradigm Shift Is Needed to Ameliorate the Ongoing Opioid Epidemic

**DOI:** 10.3390/pharmacy8030154

**Published:** 2020-08-25

**Authors:** Adriana Matos, David L. Bankes, Kevin T. Bain, Tyler Ballinghoff, Jacques Turgeon

**Affiliations:** 1 Tabula Rasa HealthCare, Applied Precision Pharmacotherapy Institute, Moorestown, NJ 08057, USA; AMatos@CareKinesis.com (A.M.); DBankes@trhc.com (D.L.B.); KBain0225@gmail.com (K.T.B.); TBallinghoff@trhc.com (T.B.); 2 Biophilia, LLC, Swedesboro, NJ 08085, USA; 3 Tabula Rasa HealthCare, Precision Pharmacotherapy Research and Development Institute, Orlando, FL 32827, USA

**Keywords:** clinical decision support system, drug interaction, drug interaction software, opioid, polypharmacy, pharmacogenomics

## Abstract

Polypharmacy is a common phenomenon among adults using opioids, which may influence the frequency, severity, and complexity of drug–drug interactions (DDIs) experienced. Clinicians must be able to easily identify and resolve DDIs since opioid-related DDIs are common and can be life-threatening. Given that clinicians often rely on technological aids—such as clinical decision support systems (CDSS) and drug interaction software—to identify and resolve DDIs in patients with complex drug regimens, this narrative review provides an appraisal of the performance of existing technologies. Opioid-specific CDSS have several system- and content-related limitations that need to be overcome. Specifically, we found that these CDSS often analyze DDIs in a pairwise manner, do not account for relevant pharmacogenomic results, and do not integrate well with electronic health records. In the context of polypharmacy, existing systems may encourage inadvertent serious alert dismissal due to the generation of multiple incoherent alerts. Future technological systems should minimize alert fatigue, limit manual input, allow for simultaneous multidrug interaction assessments, incorporate pharmacogenomic data, conduct iterative risk simulations, and integrate seamlessly with normal workflow.

## 1. Introduction

The progressively worsening population health problem of opioid use disorders and the rising death rates from opioid overdoses have caused policy makers and researchers to work on strategies for optimizing opioid medication management while concurrently curtailing opioid prescriptions [1]. Clinicians are faced with the challenge of treating pain adequately to improve patients’ quality of life while trying to avert the potential of overuse, misuse, and abuse among patients who are prescribed opioids [1]. The challenge is amplified because of the complexity of pain management and the high prevalence of polypharmacy among opioid users [2,3,4]. Polypharmacy—frequently defined as the use of five or more drugs or the use of more drugs than are medically necessary [5,6]—increases the likelihood that a patient will experience a drug–drug interaction (DDI) and an associated adverse drug event (ADE) (e.g., overdose) [7,8]. Indeed, DDIs can profoundly influence an individual’s response to opioids and have been associated with increased healthcare utilization rates and expenditures [9,10]. Therefore, DDIs could be a silent contributing factor to the opioid overuse, misuse, abuse, morbidity, and mortality that have been seen in the current opioid epidemic [8]. 

Clinical decision support systems (CDSS) are a promising solution to help clinicians identify opioid-related DDIs and avoid negative consequences [11]. Research has demonstrated that, without software aids, a clinician’s ability to identify well-documented and even clinically significant DDIs is limited, if not lacking [12,13,14,15]. Unfortunately, existing systems are far from fail-safe as an abundance of evidence indicates that clinicians may still miss clinically important DDIs, particularly in patients with polypharmacy [12,13,16,17,18,19]. In light of such troubling information, this narrative review aims to explore our current understanding of opioid-related polypharmacy and subsequent DDIs with an evidence-based appraisal of the performance of CDSS used in practice to resolve opioid-related DDIs. To conclude, we will synthesize this information into an expert opinion regarding the ideal technological features that CDSS should offer to help ameliorate opioid-related DDIs. 

## 2. Opioid Users and Polypharmacy: Defining the Scope of the Problem 

Polypharmacy is a common phenomenon among adults using opioids. A recent analysis of Medicare Part D claims reported that opioid users filled an average of 52 prescriptions per year from around 10 unique drug classes, and over 20% of opioid users took more than 10 concurrent medicines [4]. Multimorbidity and visiting multiple prescribers—which are more common among opioid users compared to non-users—are the main contributors to opioid-related polypharmacy [4,20]. For instance, the same Part D analysis found that, compared to non-users, opioid users had, on average, nearly two more comorbidities. Concomitant diagnoses may often be psychiatric-related, such as major depressive disorder or bipolar disorder [4]. This can lead to central nervous system (CNS) polypharmacy, which has been defined as the use of three or more medications with psychoactive properties (e.g., benzodiazepines, antidepressants, sedative hypnotics) [2,21]. Gerlach et al. found that 75% of older outpatients with CNS polypharmacy were opioid users [2]. This type of polypharmacy is of particular concern for older adults, since it has been linked with a higher risk of falls, cognitive impairment, accidental overdose, and mortality [22,23,24,25]. Black-box warnings regarding such co-prescribing have been explicitly issued by the US Food and Drug Administration [26]. Additionally, several screening tools exist to aid clinicians in identifying potentially inappropriate medication use, such as the 2019 Beers Criteria by the American Geriatric Society and the Screening Tool of Older Persons’ potentially inappropriate Prescriptions/Screening Tool to Alert to Right Treatment (STOPP/START) criteria [21,27]. For example, the Beers Criteria recommend avoiding the combination of opioids and benzodiazepines due to the increased risk of overdose [21].

## 3. Polypharmacy and Drug Interactions: Explaining Their Relationship

Clinicians must expect drug interactions to occur whenever polypharmacy is encountered. Research has clearly demonstrated that the occurrence of DDIs escalates as the number of drugs taken by a patient increases [6,7,28]. For instance, in a prospective cohort study of hospitalized older adults taking five or more drugs, the frequency of potential cytochrome P450 (CYP)-mediated DDIs was 80%. The probability of at least one significant DDI increased with the number of drugs taken. Specifically, significant DDI probability was 50% in a patient taking five to nine drugs and increased to 100% when a patient was taking 20 or more drugs [7].

Therefore, polypharmacy may explain the high prevalence of DDIs seen among opioid users. Estimates suggest that approximately 30% of opioid users are exposed to clinically significant interactions [29,30]. Moreover, opioids are among the most frequently prescribed medications involved in DDIs [31,32]. For example, a recent study found that 25% of all pharmacist-identified pharmacokinetic drug interactions involved opioid therapy in medically complex older adults with polypharmacy (around 16 medications per patient) [32]. 

## 4. Opioid-Related DDIs: Mechanisms and Consequences

There is a high prevalence of polypharmacy and DDIs among opioid users, and multiple mechanisms can simultaneously drive these DDIs [8,33]. In general, there are two broad categories of opioid-related DDIs: pharmacodynamic (PD) and pharmacokinetic (PK) DDIs. 

### 4.1. Pharmacodynamic

PD DDIs often involve excessive CNS depression when opioids are combined with other CNS-depressing drugs (e.g., benzodiazepines, antidepressants) [34]. Overall, PD interactions largely result from CNS polypharmacy and, as previously mentioned, can be directly life-threatening (e.g., death due to respiratory depression) or life-altering (e.g., injurious falls) and have necessitated black-box warnings. 

As a relevant example, consider the aforementioned interaction between opioids and benzodiazepines. Opioids are µ-opioid receptor agonists, which provides their analgesic effect. Benzodiazepines are gamma-aminobutyric acid (GABA) receptor agonists, which provides their sedative and anxiolytic effects. Since both the µ-opioid and GABA receptors are present on respiratory neurons, both opioids and benzodiazepines are associated with causing respiratory depression [35]. Specifically, opioids cause respiratory depression by inhibiting the µ-opioid receptors in the brainstem, whereas benzodiazepines reduce the lung’s tidal volume. Additionally, both opioids and benzodiazepines cause obstructive apneas and hypopneas via reduction in upper airway capacity [35]. Interestingly, several reports have indicated that sleep disorders or symptoms, including sleep apnea and snoring, should be given considerable attention in patients treated with opioids and/or benzodiazepine, as they may indicate impending upper airway obstruction in a sedated patient [36,37,38].

PD DDIs can also involve the modulation of neurotransmitter activity in the CNS (e.g., dopamine, serotonin) [33]. For example, tramadol is a µ-opioid receptor agonist and a serotonin and norepinephrine reuptake inhibitor. When taken at high doses (e.g., overdose), tramadol can induce serotonin release, potentially resulting in serotonin syndrome, which may be life-threatening. Additionally, combined use of tramadol and other serotonergic drugs (e.g., selective serotonin reuptake inhibitors) has been shown to cause serotonin syndrome resulting from synergistic serotonergic activity [39]. 

### 4.2. Pharmacokinetic

PK interactions involving opioids are more complex as several of them involve different isoforms of the cytochrome P450 system (CYP450). CYP450s, especially CYP2D6- and CYP3As-mediated pathways, are responsible for either activating opioid prodrugs or eliminating opioid parent drugs [35,40]. 

#### 4.2.1. CYP2D6

The most commonly prescribed opioids—codeine, tramadol, oxycodone, and hydrocodone [41,42,43]—are prodrugs metabolized by the CYP2D6 isoform into active metabolites that are significantly more potent and have greater affinity for the μ-opioid receptor compared to their parent drugs (morphine, O-desmethyltramadol, oxymorphone, and hydromorphone, respectively) [44,45,46]. CYP2D6 activity is genetically determined, with individuals exhibiting a poor (non-functional), intermediate, normal, or ultra-rapid metabolizer phenotype [47]. Patients who are CYP2D6 poor metabolizers taking prodrug opioids have demonstrated reduced pain control and augmented opioid-related adverse effects [35,44,,48,49,50,51,52]. These prodrug opioids are substrates with weak affinity for the CYP2D6 isoform; hence, they are vulnerable both to competitive inhibition by stronger-affinity CYP2D6 substrates (e.g., propranolol) and to non-competitive inhibition interactions by CYP2D6 inhibitors (e.g., quinidine, terbinafine, amiodarone) [53,54]. 

These DDIs depress CYP2D6 activity by significantly reducing conversion of prodrug opioids into their more potent active metabolites, effectively mimicking a poor metabolizer phenotype. This process is known as phenoconversion [46,51,55,56]. Indeed, lower active metabolite plasma concentrations and reduced effectiveness have been observed in PK studies and clinical trials [49,51,52,55,56,57].

Reduced analgesic effectiveness can lead to increased opioid utilization through higher dosages or increased frequency of administration [8]. Either could result in tolerance, misuse, dependence, abuse, or overdose-related death [8,58,59,60,61]. Alternatively, introducing a new drug into a patient’s opioid regimen that causes a DDI can lead to a sudden inability to form the active metabolite, which may even precipitate withdrawal in patients who are opioid dependent [62].

#### 4.2.2. CYP3A4/5

CYP3A4/5 isoforms are also involved in the metabolism of prodrug opioids and non-prodrug opioids (e.g., fentanyl); yet, studies have not consistently linked altered opioid response with modified CYP3A4 activity [44,50]. However, *CYP3A5* polymorphisms may alter the plasma concentrations of an opioid’s metabolite [35,63]. For example, patients who express CYP3A5 (i.e., carriers of the CYP3A5*1 variant) were reported to have higher plasma concentrations of noroxycodone, oxycodone’s predominant metabolite, compared to non-expressers of CYP3A5 (i.e., carriers of the CYP3A5*3 variant), resulting in a higher incidence of dose escalation [63]. Additionally, prodrug opioids are substrates with weak affinity for CYP3As and are vulnerable to non-competitive inhibition interactions by CYP3A inhibitors (e.g., ketoconazole, voriconazole, clarithromycin) and by CYP3A inducers (e.g., phenytoin, rifampin), which have been shown to increase opioid-related adverse reactions and decrease opioid response, respectively [33,64].

## 5. Current State of CDSS for Opioid DDI Management

The aforementioned DDI consequences are avoidable with proper recognition of interacting drugs followed by appropriate clinician interventions [11,12]. Advanced technology has led to the development of CDSS that can aid clinicians with the detection of DDIs and of many other risks associated with prescription opioid use [1]. 

**Figure 1:**
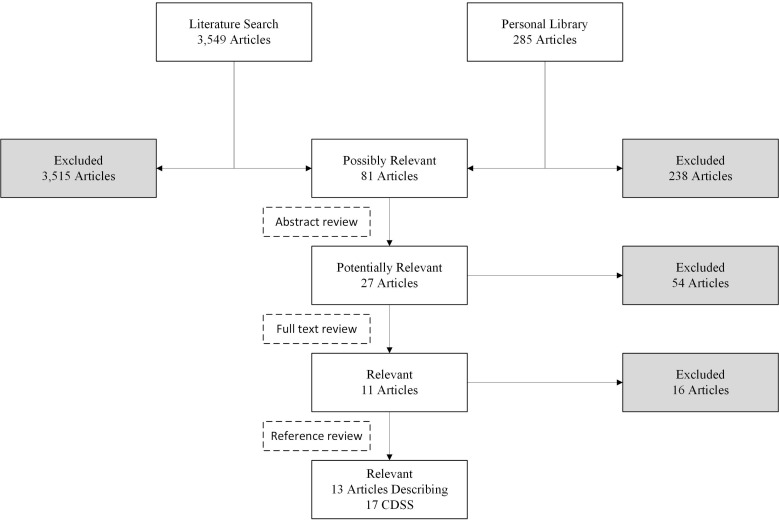
Results of search for relevant articles.

Since an abundance of literature indicates that, in general, CDSS can positively influence clinician performance and patient outcomes [1,65,66], we conducted a literature search to examine how opioid-specific CDSS address DDIs. Since non-software decision support tools (e.g., predictive models, bedside scoring algorithms) theoretically could be implemented into technology, we also included papers describing such tools in our analysis. The initial review of the evidence-based condition commenced with a thorough personal electronic library search for potentially relevant articles. This search yielded 285 articles of interest. Next, a MEDLINE search was conducted using the key search term “opioid” in combination with each of the following search terms: “clinical aid,” “clinical decision making,” “clinical decision support,” “clinical decision support system,” “clinical decision support software,” “clinical decision support tool,” “decision making,” “decision support,” “decision support system,” “decision support software,” “decision support technique,” “decision support tool,” “drug interaction alert,” “software,” “support system,” “support tool,” “system,” and “tool.” The literature search was truncated to articles published in English since 2015. In total, 3549 articles were identified via the literature search. The researchers reviewed the titles of all articles identified and excluded duplicates and articles that did not clearly focus on CDSS for opioid medication management. Of the possibly relevant articles, the researchers sequentially reviewed article abstracts, retaining those that were potentially relevant, and then the full text of those remaining. Using this methodology, 11 relevant articles were identified (Figure 1). The researchers identified two additional articles for this review by hand-searching references of the retained articles. In total, these 13 references described 17 CDSS that were included for further analysis.

While economic evaluations (e.g., return-on-investment, cost-benefit analysis) are vital for healthcare systems to consider when adopting new technology or CDSS, our primary focus was to analyze the clinical and system-related content of CDSS. We detail the features of each individual tool identified from the literature search in Table 1 as well as summarize opioid-specific CDSS features and shortcomings in Table 2 [1,19,67,68,69,70,71,72,73,74,75,76,77,78,79,80,81,82,83,84,85]. Examples of the systems that we encountered are OpioidCalc NYC, the Safe Opioids application, opioid pain medication documentation, and surveillance system. We found that opioid-specific CDSS often featured opioid prescription aides, conversion calculators, drug alerts, prescribing guidelines, and pain assessment tools; however, DDIs were assessed by embedding general (i.e., non-opioid specific), commercially available drug interaction software (e.g., First Databank) [19] or focused on very specific interactions (e.g., sedative or benzodiazepine co-prescribing) [72,80]. In addition, we noticed several shortcomings of these CDSS which can be categorized as system- or content-related. A common system-related shortcoming of CDSS is requiring clinicians to manually input patient-specific information (e.g., drug regimen, opioid usage) that could ideally be obtained automatically from electronic health records (EHRs). A common content-related shortcoming is failing to include other contextual information that could potentially affect opioid drug interactions, such as pharmacogenomic (PGx) test results. 

Given that several opioid-specific CDSS do not directly or comprehensively address DDI, clinicians prescribing opioids must rely on general software for a comprehensive DDI assessment. Therefore, the shortcomings of these systems must be highlighted. Specifically, studies have consistently shown that drug interaction software frequently elicits alert fatigue and dismissal, misses clinically important interactions, and is unable to assess simultaneous multidrug interactions [7,12,40,85,86,87,88,89]. Indeed, most systems analyze drug interactions in a one-drug-to-one-drug manner and do not consider the entire drug regimen in their analysis. This is a significant shortcoming as several opioid users present with polypharmacy. Similar to the opioid-specific CDSS, few systems integrate PGx data; therefore, the software fails to detect patient-specific drug–gene and drug–drug–gene interactions [40,85,86]. Table 3 summarizes the shortcomings that are common to most commercially available drug interaction alert software programs and also describes content- and system-related ideal characteristics of both stand-alone and CDSS-integrated drug interaction alert software [1,11,12,40,75,89,90]. 

## 6. Features of an Optimal Opioid CDSS: An Expert Opinion with a Case Discussion

The complexity of opioid-related drug interactions requires a multifaceted technological overhaul to address the identified content- and system-related shortcomings. In addition to being readily accessible, user friendly, on-demand/timely, integrated into workflows, interactive with medical and drug information, patient-specific, up-to-date, and secure, we believe that there are three critical features that have not been explored as extensively in the literature. We believe that these features can address many concerns related to opioid-involved DDIs resulting from polypharmacy.

### 6.1. Content-Related Consideration: Embrace Simultaneous Assessments with a Comprehensive Visualization of Pertinent DDI Mechanisms

Unfortunately, the overwhelming majority of CDSS analyze DDIs in a one-drug-to-one-drug, pairwise manner [11,40]. For patients with polypharmacy, pairwise analysis often generates multiple incoherent results, leaving clinicians uninformed about the most probable and serious consequence(s) of the interaction(s) and unsure of the precise intervention(s) needed to appropriately mitigate the interaction(s) [11]. An ideal opioid CDSS should include drug interaction alert software that is capable of analyzing simultaneous multidrug interactions and comprehensively describing the mechanisms and severities underlying the interactions. While the Medication Risk Mitigation (MRM) Matrix™ is not an opioid-specific CDSS, it is one example of a general CDSS with features that considers polypharmacy, alert fatigue, and the multimechanistic nature of opioid-related DDIs [7,28,32,91]. 

**Figure 2:**
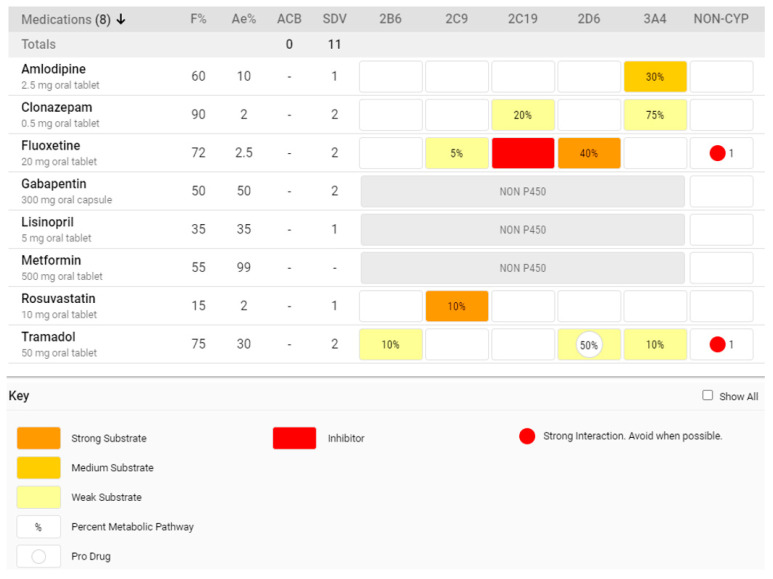
Medication regimen utilizing the Medication Risk Mitigation (MRM) Matrix™.

Figure 2 illustrates how the MRM Matrix^™^ allows users to simultaneously view all profiled medications and their relevant PK and PD mechanisms without generating any alerts. In the hypothetical case depicted, it can be seen that this patient taking tramadol is suffering from overall polypharmacy as well as CNS polypharmacy (clonazepam, tramadol, gabapentin, and fluoxetine). As such, we see a high CNS sedative burden score (as measured by the Sedative Load Model [92]), indicating that this individual is at particularly high risk of respiratory depression associated with opioid therapy. Additionally, from a PD standpoint, serotonergic toxicity is enhanced due to the presence of fluoxetine [93] and is denoted by the red circle in the “non-CYP” column. PK interactions compound these risks and create new problems. Firstly, we see that the individual is likely experiencing higher than expected blood concentrations of clonazepam [94]—augmenting the overall CNS burden—due to competitive inhibition with amlodipine showing greater affinity (darker yellow) for CYP3A4 than clonazepam (pale yellow) [95,96]. Secondly, we see that tramadol, a prodrug opioid, is suffering from competitive inhibition by fluoxetine. Therefore, tramadol’s active metabolite, O-desmethyltramadol (M1), is not formed and the patient is likely experiencing inadequate analgesia. This can lead to escalating tramadol doses [8,97,98]. Moreover, higher concentrations of the parent drug tramadol can further worsen CNS depression, increase risks for serotonergic toxicity, and reduce the seizure threshold [39]. 

The hypothetical case above illustrates that a coherent assessment of opioid-involved DDIs is possible through a comprehensive visualization of pertinent pharmacology. Clinicians do not necessarily need to be “alerted” in a pairwise manner. This can have a profound effect on the medication safety review process. For example, pharmacists are likely to identify more drug interactions when using this CDSS as compared with pairwise alert software [7,28,32]. Moreover, studies assessing the aforementioned CDSS have shown that opioids are among the most common drug classes involved in identified DDIs among older adults with polypharmacy [32,91]. 

### 6.2. Content-Related Consideration: Simulate, Quantify, and Estimate Risk Associated with the Interactions Present in the Current Regimen

An ideal drug interaction alert software for opioid management should allow clinicians to perform iterative drug regimen simulations to aid with patient-specific decisions about alternative drugs that avert or mitigate interactions with opioids and other drugs. Simulations should attempt to quantify changes to an individual’s overall risk of ADEs using risk prediction models [99,100]. 

To illustrate these points, there are several prudent alterations that a clinician could consider in order to reduce risks associated with the tramadol interactions in the case above. Firstly, the patient could switch from fluoxetine to venlafaxine. While this will not eliminate serotonergic toxicity concerns, it will mitigate the severity of the interaction occurring with tramadol at CYP2D6. Theoretically, this will allow tramadol to work optimally and result in lower or less frequent opioid dosing. In addition, since venlafaxine may be useful for the treatment of neuropathic pain [101], this antidepressant switch could provide an opportunity to slowly reduce the dose of gabapentin, with the goal of complete gabapentin discontinuation. If accomplished, this would help reduce the overall CNS burden. Next, if benzodiazepine therapy is absolutely needed, the clinician could consider switching from clonazepam to lorazepam, which is not metabolized by the CYP450 system and will not interact with amlodipine, thereby reducing the overall CNS burden. 

**Figure 3:**
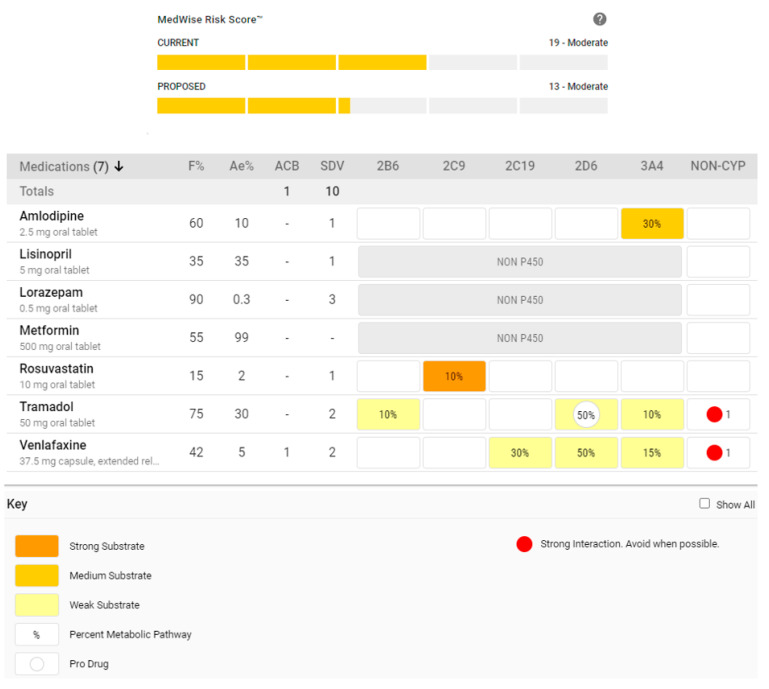
Amended medication regimen utilizing the Medication Risk Mitigation (MRM) Matrix™ and displaying the MedWise Risk Score™ (MRS).

The hypothetical drug regimen profile, with these suggested amendments, is illustrated in Figure 3. The MedWise Risk Score™ (MRS), which is an ADE risk prediction tool embedded in the MRM Matrix™ CDSS, has decreased by six points. In medically complex older adults, each point increase in the MRS is associated with nearly a 10% increase in the odds of ADEs and an additional $1000 USD spent on medical therapy [100]. Therefore, it can be expected that addressing these drug interactions with the aforementioned interventions can mitigate opioid-related risks, enabling the clinician to feel more confident making changes. 

### 6.3. System-Related Consideration: EHR Interoperability

While CDSS provide clinicians with useful tools for guiding the decision-making process and should not replace the need for clinical assessment by clinicians when optimizing the use of opioids, many clinicians dislike mandated usage of yet another system [1]. Utilizing a standardized data exchange model is of the utmost importance. When there are no accredited standards, CDSS applications would need to be developed and rewritten in order to “fit” into each hospital’s EHR system (e.g., Allscripts, Cerner, and Epic), which poses challenges for developers and is not feasible or sustainable. Therefore, providing an open access and interoperability technology platform that can interface with different healthcare system EHRs should be the goal [1,102]. This enables clinicians to easily and efficiently access a library of CDSS applications to securely access EHR data in order to improve opioid medication management [1]. These platforms can also be used to give patients access to their health data as well as input personal data and measures of functional status and pain control, in order for patients to participate in their pain management and overall healthcare with clinicians. In the US, there are currently three health information technology standards leading healthcare interoperability (e.g., Direct, Fast Health Interoperability Resources (FHIR) and cloud fax) [103]. Such systems have developed the capability to include genomic data standards [1]. As a result, incorporating PGx information into CDSS unifies PGx data from multiple vendors. Thus, CDSS applications for opioid medication management that follow a standardized model could seemingly be used by any healthcare institution or provider equipped with these systems [1]. 

## 7. Conclusions

Polypharmacy is common among opioid users. This phenomenon can lead to complex, multimechanistic drug interactions that can potentially cause a plethora of serious and life-threatening consequences. While CDSS have the potential to help clinicians better manage drug interactions arising from polypharmacy, existing evidence suggests that both opioid-specific CDSS and general DDI software have several system- and content-related limitations that need to be overcome. Future technological enhancements should attempt to minimize alert fatigue, allow for simultaneous multidrug interaction assessments, incorporate PGx data, conduct iterative risk simulations, and integrate seamlessly with clinician workflow. The MRM Matrix™ is a promising CDSS that incorporates these enhancements, and incorporating a standardized data exchange platform is a promising solution to be used by EHRs without causing disruption to clinicians’ workflow.

## Figures and Tables

**Table 1:** Overview of features from clinical decision support systems.

Source	Country	Setting	Sample Size, N	Name of CDSS	Feature(s) of CDSS
Blum, 2018 [68]	Multinational	N/A	N/A	Genetic Addiction Risk Score™ (GARS)	Risk score based on a panel of genes and polymorphisms associated with addiction risk and associated behaviors
Brenton, 2017 [69]	US	24 study sites	908	Proove Opioid Risk (POR) algorithm	Risk score based on genomic testing Genetic markers include 11 single-nucleotide polymorphisms implicated in opioid misuse or abuse Phenotypic factors include age, personal history of alcohol or substance abuse, and a history of various metal health diagnoses Scores ≥24 indicate high risk of opioid use disorder
CDC [70]	US	N/A	N/A	CDC Opioid Guideline App	Calculates MME Provides brief synopses of guideline recommendations Includes a glossary Overview of motivational interviewing techniques Links to full CDC guideline with references
Christ, 2018 [71]	US	University of Chicago Medicine	30 (pre-enactment); 32 (post-enactment)	Pain Clinical Decision Support Tool (PCDST)	Prioritizes patients based on most recent pain score Includes chart of laboratory values and vital signs (e.g., respiratory rate, heart rate) Adheres to National Comprehensive Cancer Network clinical guideline
Genco, 2016 [19]	US	ED	4581	Epic electronic health record and computerized provider order entry system (Epic Systems Corporation, Verona, WI) with the First Databank drug information plug-in (First Databank, Inc., San Francisco, CA)	Opioid alerts (e.g., drug-drug interactions) – interruptive alerts resulting in a pop-up and requiring clinician response
Malte, 2018 [72]	US	Veteran Affairs healthcare system	1332	No name provided	EHR point-of-prescribing alert Triggers when opioids and benzodiazepines are co-prescribed
Maurer, 2016 [73]	US	N/A	N/A	Safe Opioids application	Assessment of pain using the Opioid Risk Tool with links to PDMP Links to medical guidelines Includes list of common side effects Provides guidance on how to talk to patients about opioid abuse Includes references
NYC Department of Health and Mental Hygiene [74]	US	N/A	N/A	OpioidCalc NYC	Calculates MME Alerts for high dosages of opioid(s) Includes references
Oliva, 2017 [75]	US	Veteran Affairs healthcare system	1,135,601	StratificationTool for Opioid Risk Mitigation (STORM)	Prioritizes patients for review and intervention according to their modeled risk for overdose/suicide-related events Displays risk factors and risk mitigation interventions Includes EHR-data extracts
Patel, 2018 [76]	US	Veteran Affairs healthcare system	7602	Chronic Opioid Therapy–Clinical Reminder (COT-CR)	Identifies chronic opioid users by alerting clinicians (i.e., clinical reminder) Prompts clinicians to document reason for chronic opioid therapy Guides clinicians through a 3-part pain assessment
Philip Eagan [77]	US	N/A	N/A	pH-Medical Opioid Converter App	Calculates MME Opioid to opioid conversion calculator Links to guidelines
Ponton, 2018 [78]	UK	41 general practitioner practices	1881	No name provided	Electronic spreadsheet tool Calculates MME Triggers alert for patients taking ≥120 mg of MME Relies on manual data entry
Price-Haywood, 2018 [79]	US	Primary care providers, Ochsner Health System	2640	Opioid Risk Tool (ORT)	Risk factors associated with substance abuse (e.g., personal and family history of substance abuse) Risk stratification for opioid-aberrant behavior Annual pain contract Functional assessment Links to several resources (e.g., CDC guideline fact sheet)
Sinha, 2017 [1]	US	N/A	N/A	Substitutable Medical Applications and Reusable Technologies (SMART) for CDSS app development	Open source standard Third-party capability to develop an application for accessible and efficient implementation into EHR
Soto, 2015 [80]	US	Inpatient	N/A	Michigan Opioid Safety Score (MOSS)	Manual scoring tool that assesses inpatient risks of opioid-related ADEs Administered prior to further opioid use Scores range from 0 (safe to proceed with further opioid dosing) to 4 (decrease opioid use), with a “Stop” modifier (based on Pasero Opioid-Induced Sedation Scale) Scores incorporate pertinent patient risks (e.g., age, body mass index, type of surgery, anesthesia time, sedative use within 2 h) and respiratory rate
Trafton, 2010 [81]	US	Veteran Affairs healthcare system	N/A	No name provided	Computerized decision support system that operationalized the 2003 Veterans Administration/Department of Defense clinical practice guidelines for opioid use in chronic non-cancer pain Rules engine/algorithm provides clinicians with guideline-driven recommendations (e.g., dosing, titration, warnings, contraindications)
Wilsey, 2009 [82]	US	Veteran Affairs Pain Clinic	1400	The Prescription Opioid Documentation and Surveillance (PODS) System	Computer-assisted survey administration instrument Algorithmically assesses addiction risks and level of pain control Surveys consist of self-administered medical (e.g., depression screenings), substance abuse (e.g., Screener and Opioid Assessment for Patients with Pain (SOAPP®)), and pain management (e.g., pain catastrophizing scale)

Abbreviations: ADEs = adverse drug events; CA = California; CDC = Centers for Disease Control and Prevention; CDSS = clinical decision support system; ED = emergency department; EHR = electronic health record; MME = morphine milliequivalent; N/A = not applicable; NYC = New York City; PDMP = prescription drug monitoring program; UK = United Kingdom; US = United States; WI = Wisconsin.

**Table 2:** Features and shortcomings of most commercial CDSS involving opioid medication management ^1^.

**Common Features**	**Description**
Opioid prescription aides	Guiding the practice of prescribing opioids, such as quantity and days’ supply limitations.
Opioid conversion calculators	Determining the equianalgesic dose between opioids by calculating the total daily MME, taking into consideration the specific opioid, strength, and quantity.
Opioid drug alerts	Alerting clinicians to opioid-related factors that may pose a risk to the patient. Opioid drug–allergy interaction Opioid drug–drug interaction Opioid duplicate therapy High-dose opioid therapy (e.g., MME/day ≥90) UDS results
Opioid prescribing guidelines	Referencing clinical practice guidelines to assist clinicians with opioid medication management.
Pain assessment tools	Utilizing applications and/or scoring methods for assessing the patient’s pain.
**Common Shortcomings**	**Description**
System-related	Requiring extensive training or being time-consuming to use. Lacking clinician involvement in feature development. Requiring internet access or separate login, independent of the EHR. Requiring the internet to download clinical practice guidelines and other resources rather than having them embedded in the CDSS. Requiring manual input of data and/or patient-specific information or having many plug-in values that could be automatically taken from the EHR. Disrupting or not efficiently integrating with clinician workflow. Lacking the ability to mine and utilize free text from the EHR (e.g., treatment plan notes).
Content-related	Lacking information about patient-clinician shared decision-making, particularly treatment goals and preferences (e.g., palliative care, pain management). Under-reporting of drug- or opioid-related adverse events (e.g., falls, overdoses) for consideration in decision-making. Lacking the ability to distinguish true allergies or hypersensitivity reactions from pseudo-allergies or opioid-related side effects (e.g., itching) and intolerances (e.g., nausea). Lacking actionable mitigation strategies and recommendations (e.g., using alternative drugs or opioids, tapering opioids, changing times of administration). Over alerting clinicians to all information, instead of flagging information of highest clinical importance. Having outdated or no citations for evidence-based assessment or lacking access to reference literature. Failing to integrate PGx data. Missing information about other high-risk conditions (e.g., mental health, substance use disorder) that may compromise optimal opioid medication management.

Abbreviations: CDSS = clinical decision support system; EHR = electronic health record; MME = morphine milliequivalent; PGx = pharmacogenomics; UDS = urine drug screening. ^1^ Information compiled from numerous references [1,19,67,68,69,70,71,72,76,83,84,85,86].

**Table 3:** Shortcomings and ideal characteristics of drug interaction alert software ^1^.

**Shortcomings of Drug Interaction Alert Software**	
**System-related**	Over alerting of DDIs with potentially low clinical relevance, creating “alert fatigue” and dismissal of alerts as well as being time-consuming and possibly mentally draining. Under-detecting of clinically significant drug interactions. Largely accounting for DDIs that have been reported or (more accurately) published while failing to account for theoretical interactions based on established pharmacological properties. Disrupting or inefficient integration within clinicians’ workflow.
Content-related	Detecting only one-to-one drug interactions, which often generates numerous incoherent results, especially in patients with polypharmacy. Lacking consideration for the pharmacological mechanisms causing or contributing to the opioid-involved DDI. Lacking differentiation between PD and PK interactions. Lacking severity ratings for drug interactions, leaving clinicians to discern clinical importance. Lacking integration of PGx data and therefore assessment of DGIs and DDGIs involving opioids. Missing reference literature or having no citations to substantiate the nature and severity of DDIs.
**Ideal Characteristics of Drug Interaction Alert Software**	
System-related	Interfacing with different healthcare systems and pharmacies to present the most complete and accurate information about all drugs in a patient’s regimen. Interacting with PGx information to assist in identifying DGIs, DDGIs, and phenoconversions. Performing a single, simultaneous, multidrug assessment rather than multiple, sequential, two-drug assessments for patients with polypharmacy. Discerning interactions based on active ingredient (for combination products) and route of administration. Undergoing regular (e.g., monthly) updates. Identifying all clinically relevant drug interactions (high sensitivity) without mistakenly alerting the clinician over interactions that are dubious: low risk or low clinical relevance (high specificity). Using fast, modern, interactive technology that uses APIs for integrated CDSS development. Standardizing terminology and vocabulary (e.g., SNOMED CT allows clinicians to uniformly record drug interaction findings). Using machine learning to make successful predictions about DDIs based on past experiences.
Content-related	Displaying only pertinent information to prevent overburdening the user with clinically insignificant alerts. Constructing recommendations to mitigate DDIs based on predefined algorithms and rules (e.g., auto-generate appropriate alternative drug(s) to mitigate opioid-involved DDI). Requiring sound clinical judgment. Including information and mechanisms underlying PD, PK, and PGx interactions. Quantifying the magnitude of expected changes in plasma drug concentrations. Providing context about the severity of the DDI effect. Providing rapid access to up-to-date information supporting mechanisms and clinical significance of identified drug interaction. Allowing interactive simulations with other drugs or virtual testing environments to aid clinicians with decisions about alternative, non-interacting drugs.

Abbreviations: APIs = application programming interface; CDSS = clinical decision support systems; DDGI = drug–drug–gene interaction; DDI = drug–drug interaction; DGI = drug–gene interaction; PD = pharmacodynamic; PGx = pharmacogenomics; PK = pharmacokinetic; SNOMED CT = Systematized Nomenclature of Medicine—Clinical Terms. ^1^ Information compiled from numerous references [1,11,12,40,75,89,90].
